# Phytoremediation of Meta-Cresol by Sunflower: Tolerance of Plant and Removal of M-Cresol

**DOI:** 10.3390/toxics13100845

**Published:** 2025-10-03

**Authors:** Hui Li, Shuai Su, Yujia Jiang, Hong Chen, Liudong Zhang, Yi Li, Shengguo Ma, Jiaxin Liu, Haitao Li, Degang Fu, Kun Li, Huicheng Xie

**Affiliations:** 1 College of Agriculture and Forestry Science, Linyi University, Linyi 276000, China; huili@lyu.edu.cn (H.L.); 17662880205@163.com (S.S.); 2 Shandong Provincial Forestry Protection and Development Service Center, Jinan 250014, Chinazhangliudong@shandong.cn (L.Z.); mshguo@163.com (S.M.); 3 College of Forestry, Shandong Agricultural University, Taian 271018, Chinaliujxin0622@163.com (J.L.); kunli@sdau.edu.cn (K.L.); 4 State-Owned Forest Farm Headquarters of Feixian, Feixian 273400, China; 15753910969@163.com

**Keywords:** M-cresol stress, phytoremediation function of sunflowers, physiological response, chlorophyll fluorescence, removal of m-cresol

## Abstract

Meta-cresol (m-cresol) is highly corrosive and toxic, and is widely present in industrial wastewater. As a pollutant, it adversely affects various aspects of human production and daily life. To evaluate the feasibility of using sunflowers to remediate m-cresol-contaminated wastewater, this study used *Helianthus annuus* L. as the test subject to analyze its tolerance and the wastewater purification efficiency under different m-cresol concentrations. The results showed that the net photosynthetic rate (*P*_n_), transpiration rate (*T*_r_), stomatal conductance (*G*_s_), and light energy utilization efficiency (LUE) of Helianthus annuus L. exhibited an overall decreasing trend, while the intercellular CO_2_ concentration (*C*ᵢ) initially increased and subsequently decreased with increasing m-cresol concentration. When m-cresol concentration reached or exceeded 60 mg·L^−1^, the net photosynthetic rate and intercellular CO_2_ concentration in the leaves showed opposite trends with further increases in m-cresol stress. The inhibition of net photosynthesis in sunflowers by m-cresol was mainly attributed to non-stomatal factors. The maximum photochemical efficiency (*F*_v_/*F*_m_), actual photochemical efficiency (ΦPSII), photochemical quenching coefficient (*q*^P^), PSII excitation energy partition coefficient (α), and the fraction of absorbed light energy used for photochemistry (P) all decreased with increasing m-cresol concentration. In contrast, non-photochemical quenching (NPQ), the quantum yield of regulated energy dissipation [Y(NPQ)], and the fraction of energy dissipated as heat through the antenna (D) first increased and then decreased. Under low-concentration m-cresol stress, sunflowers protected their photosynthetic system by dissipating excess light energy as heat as a stress response. However, high concentrations of m-cresol caused irreversible damage to Photosystem II (PSII) in sunflowers. Under m-cresol stress, chlorophyll a exhibited strong stability with minimal degradation. As the m-cresol concentration increased from 30 to 180 mg·L^−1^, the removal rate decreased from 84.91% to 11.84%. In conclusion, sunflowers show good remediation potential for wastewater contaminated with low concentrations of m-cresol and can be used for treating m-cresol wastewater with concentrations ≤ 51.9 mg·L^−1^.

## 1. Introduction

Phenolic compounds are major raw materials for the chemical industry. M-cresol (C_7_H_8_O), which is widely used in the production of dyes, organic pesticides, perfumes, pharmaceuticals, plastics, phenolic resins, etc. [1,2], has been classified as a “priority control substance” by the US EPA due to its difficult biodegradability and biotoxicity [3]. It has also been listed as a priority pollutant by the U.S. EPA [4,5]. M-cresol is a prototypical qualitative toxicant that is toxic to all living organisms. It is more toxic than phenol and more difficult to degrade than both o-cresol and p-cresol [6]. M-cresol cannot be efficiently removed by traditional activated sludge methods in wastewater treatment plants and has been detected in both municipal wastewater treatment facilities and receiving water environments [7]. The presence of m-cresol may significantly impact the survival and reproduction of flora and fauna in receiving water basins [8]. When its concentration in water exceeds 0.5 mg·L^−1^, it may cause damage to the human liver and digestive system due to its toxicity [9,10].

Phytoremediation is a cost-effective technology that can be described as using living plants as solar-powered pumps, which extract and concentrate specific elements from the environment. Plants are used to uptake, adsorb, or absorb inorganic and organic pollutants from soil, water, sediments, and possibly air [11]. Phytoremediation not only removes pollutants but also promotes the recycling of nutrients in wastewater, greens the land, improves the regional climate, and facilitates eco-friendly recycling [12]. In conclusion, it is a low-cost and environmentally friendly technology. Zhao found that *Salix matsudana* can be used to purify wastewater containing phenol at concentrations lower than 150 mg·L^−1^ [13]. Fu found that *Acorus calamus* was effective in removing phenol at concentrations ≤ 200 mg·L^−1^ within 21 days of treatment [14]. Liu used ice plant (*Agropyron cristatum* L. Gaertn.) in a phytoremediation experiment on aniline-contaminated soil and found that its roots could absorb aniline and translocate it through the stems to the leaves and spikes [15]. After in situ phytoremediation using indigenous herbs, heavy metal and metalloid levels in the wastewater were reduced by nearly 60% [16].

Sunflower (*Helianthus annuus* L.) is an annual herb with economic and ornamental value, characterized by strong low-temperature and drought tolerance [17] as well as self-healing ability [18]. It is known for its strong enrichment capacity for heavy metals [19,20] and has demonstrated potential in remediating lead-contaminated soil [21]. Studies have shown that oilseed sunflower exhibits higher tolerance and remediation efficiency than cotton in cadmium-contaminated soils, making it a preferable choice for phytoremediation in such areas [22]. Low concentrations of aniline stress (<100 mg·L^−1^) were found to promote sunflower growth, indicating its strong enrichment and degradation capacity for aniline at low levels [23]. A study by Jha et al. reported that sunflower hairy roots achieved 99% removal of phenol at a concentration of 100 mg·L^−1^ after 144 h of incubation [24]. Therefore, sunflower possesses a sound basis for environmental adaptation and significant potential for remediating m-cresol-contaminated wastewater. In this study, we investigated the effects of different m-cresol concentrations on sunflower growth, chlorophyll fluorescence parameters, chlorophyll content, and photosynthetic performance, as well as its purification efficiency, to evaluate the potential of sunflower for the phytoremediation of m-cresol.

## 2. Materials and Methods

### 2.1. Experimental Design

The hydroponic experiment was conducted using American oil sunflower (*Helianthus annuus* L.) No. 1 at the laboratory of Shandong Agricultural University (117°09′ E, 36°10′ N). Full, uniformly sized seeds were selected, soaked for 24 h, and then buried in vermiculite moistened with deionized water in a plastic box. Water every other day. After two weeks, healthy seedlings of uniform growth were selected and transferred to 250 mL conical flasks containing 250 mL of half-strength Hoagland solution (Alfa Aesar, Shanghai, China). The flasks were wrapped with black plastic bags. Water loss due to evapotranspiration was replenished with half-strength Hoagland solution every other day. After six weeks, uniformly grown sunflower seedlings were selected, and their initial fresh weight was measured. One seedling was placed in each conical flask containing 250 mL of either half-strength Hoagland’s solution only (control) or solution spiked with m-cresol (Thermo Fisher Scientific Inc., Heysham, UK) at concentrations of 30, 60, 90, 120, 150, and 180 mg·L^−1^ [25,26,27]. Each treatment was performed in triplicate. After adding the solution, the liquid level was marked, and the nutrient solution was replenished to the marked level every other day. After 8 days of stress treatment, photosynthetic gas exchange parameters, chlorophyll fluorescence, and chlorophyll content of the sunflowers were measured. The fresh weight of the sunflowers was measured again at the end of the experiment.

### 2.2. Measuring Items and Methods

#### 2.2.1. Photosynthetic Parameters and Chlorophyll Fluorescence Measurements

The Li-6800XT fully automatic portable photosynthesis system (LI-COR Inc., Lincoln, NE, USA) was used to measure photosynthetic gas exchange parameters. The net photosynthetic rate (*P*_n_), transpiration rate (*T*_r_), intercellular CO_2_ concentration (*C*_i_), and stomatal conductance (*G*_s_) of mature leaves were measured under sunny conditions. Three mature leaves from the upper and middle parts of each sunflower seedling were selected, with three replicates per treatment. Photosynthesis measurements were conducted at a CO_2_ concentration of 400 ± 10 μmol·mol^−1^, a leaf chamber air temperature of 25 °C, a relative humidity of 40–45%, and a photosynthetically active radiation level of 1200 μmol·m^−2^·s^−1^.

Some other photosynthetic parameters were calculated according to the equations as follows [28]:

$$\mathrm{Water-use}\;\mathrm{efficiency}\;(\mathrm{WUE})\;={\;P}_{n}/T_{r}$$

$$\mathrm{light}\;\mathrm{energy}\;\mathrm{utilization}\;\mathrm{efficiency}\;(\mathrm{LUE})=P_{n}/PAR$$

$$\mathrm{porosity}\;\mathrm{limit}\;\mathrm{value}\;(L_{s})=1-C_{i}/C_{a}$$

PAR: photosynthetically active radiation; *C*_a_: the air CO_2_ concentration (*C*_a_)

A pulse-modulated fluorescence monitoring system (Hansatech, King’s Lynn, Norfolk, UK) was employed to determine chlorophyll fluorescence parameters, including the minimal fluorescence yield of the light-adapted state (*F*_o_’), maximum fluorescence yield of the light-adapted state (*F*_m_’), minimal fluorescence yield of the dark-adapted state (*F*_o_), maximum fluorescence yield of the dark-adapted state (*F*_m_), and steady-state fluorescence yield (*F*_s_). Some other Chl fluorescence parameters were calculated according to the equations as follows [29]:

$$\mathrm{Maximal}\;\mathrm{quantum}\;\mathrm{yield}\;\mathrm{of}\;\mathrm{PSII}\;\mathrm{photochemistry}\;(F_{v}/F_{m})\;=\;\left(F_{m}-F_{o}\right)/F_{m}$$

$$\mathrm{Photochemical}\;\mathrm{quenching}\;\mathrm{coefficient}\;(q^{P})=\left({F_{m}}^{\prime }-F_{m}\right)/\left({F_{m}}^{\prime }-{F_{o}}^{\prime }\right)$$

$$\mathrm{Non-photochemical}\;\mathrm{quenching}\;(\mathrm{NPQ})=\left(F_{m}-{F_{m}}^{\prime }\right)/{F_{m}}^{\prime }$$

$$\mathrm{Effective}\;\mathrm{quantum}\;\mathrm{yield}\;\mathrm{of}\;\mathrm{PSII}\;\mathrm{photochemistry}\;(\Phi_{\mathrm{PSII}})=\left({F_{m}}^{\prime }-F_{s}\right)/{F_{m}}^{\prime }$$

The quantum yields of regulated energy dissipation [Y(NPQ)] and non-regulated energy dissipation [Y(NO)] were calculated according to the following equations [30].

$$Y(\mathrm{NPQ})=1-\Phi_{PSII}-1/\left[NPQ+1+q^{p}\times {F_{o}}^{\prime }/F_{s}\times \left(F_{m}/F_{o}-1\right)\right]$$

$$Y(\mathrm{NO})=1/\left[NPQ+1+q^{p}\times {F_{o}}^{\prime }/F_{s}\times (F_{m}/F_{o}-1)\right]$$

The PSI excitation energy distribution coefficient (α) and PSII excitation energy distribution coefficient (β) were calculated using the following formulas [31]:

$$f\;=\;\left({F_{m}}^{\prime }-F\right)/\left({F_{m}}^{\prime }-{F_{o}}^{\prime }\right)$$

$$\alpha =f/\left(1+f\right)$$

$$\beta =1/\left(1+f\right)$$

F: current fluorescence yield under actinic light

The proportion of absorbed luminous energy used for photochemical reactions (P), antenna heat dissipation (D), and non-photochemical dissipation (Ex) was calculated using the following formulas [32]:

$$P\;=\;\left({F_{m}}^{\prime }-{F_{o}}^{\prime }\right)/{F_{m}}^{\prime }\times q^{p}$$

$$D=1-\left({F_{m}}^{\prime }-{F_{o}}^{\prime }\right)/{F_{m}}^{\prime }$$

$$\mathrm{Ex}=\left({F_{m}}^{\prime }-{F_{o}}^{\prime }\right)/{F_{m}}^{\prime }\times \left(1-q^{p}\right)$$

#### 2.2.2. Determination of Chlorophyll Content

Chlorophyll content was determined using ethanol extraction. A 95% ethanol solution served as the blank control. Absorbance was measured at 665 nm and 649 nm using a spectrophotometer (Macklin Biochemical Technology Co., Ltd., Shanghai, China). The chlorophyll concentration was calculated according to the following formula [33]:

$$\mathrm{chlorophyll}\;a\;(\mathrm{Chl}a)\;=\;13.95\times A_{665}-6.88\times A_{649}$$

$$\mathrm{chlorophyll}\;b\;(\mathrm{Chl}b)=24.96\times A_{649}-7.32\times A_{665}$$

Total chlorophyll (Chla+Chlb)=Chla content+Chlb content

A_665_: the absorbance at 665 nm; A_649_: the absorbance at 649 nm; A_470_: the absorbance at 470 nm.

#### 2.2.3. Determination of M-Cresol

To determine m-cresol concentration, 1.0 mL of the sample solution was mixed with 5.0 mL of water. Then, the following reagents were added sequentially: pH 9.8 buffer solution. 0.3% 4-aminoantipyrine solution, 1.2% potassium ferricyanide solution, and 1.0 mL of 1 mol/L potassium dihydrogen phosphate solution. After thorough mixing, the solution was allowed to stand at room temperature for 10 min. The absorbance was then measured at 510 nm using a UV-Vis spectrophotometer (Epoch2T, Biotek, Winooski, VT, USA) according to the method described in reference [34]. The removal percentage of m-cresol (*P*_r_) was calculated using the following formula.

$$P_{r}\;(\%)=\frac{C_{o}-C_{f}}{C_{o}}\times 100\%$$

*C*_o_: initial concentration of m-cresol (mg L^−1^); *C*_f_: final concentration of m-cresol (mg L^−1^).

#### 2.2.4. Relative Growth Rate (RGR)

The relative growth rate (RGR) was calculated according to Beadle [35] as follows:

$$\mathrm{RGR}\;=\;\left(\ln W_{2}-\ln W_{2}\right)/\left(T_{2}-T_{1}\right)$$

W_1_: initial fresh weight (at time T_1_); W_2_: final fresh weight (at time T_2_); T_2_ − T_1_: time interval between measurements.

### 2.3. Statistical Analysis

A one-way analysis of variance (ANOVA) followed by Duncan’s multiple range test was performed using SPSS 26.0 (IBM, Chicago, IL, USA), with the significance level set at *p* < 0.05. Figures were plotted using Origin 2021 software (OriginLab, Northampton, MA, USA).

## 3. Results

### 3.1. Effects of M-Cresol Stress on Photosynthetic Parameters of Sunflower

**Figure 1:**
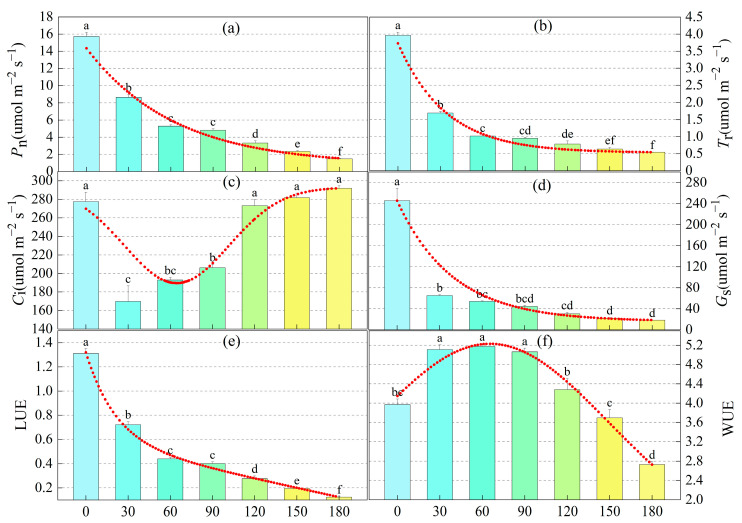
Effects of m-cresol stress on photosynthetic parameters of sunflower. (**a**) Net photosynthetic rate (*P*_n_); (**b**) Transpiration rate (*T*_r_); (**c**) Intercellular CO2 concentration (*C*_i_); (**d**): Stomatal conductance (*G*_s_); (**e**) Light energy Utilization efficiency (LUE); (**f**) Water utilization efficiency (WUE). Note: The red line indicates the trend lines of *P*_n_, *T*_r_, *C*_i_, *G*_s_, LUE and WUE with increasing m-cresol concentration; Different colors represent different concentrations of m-cresol; Different lowercase letters mean a significant difference among treatments at *p* < 0.05.

M-cresol inhibited Net photosynthetic rate (*P*_n_), Transpiration rate (*T*_r_), and Stomatal conductance (*G*_s_) at certain concentrations, while intercellular CO_2_ concentration (*C*_i_)decreased initially and then increased (Figure 1). At m-cresol concentrations ranging from 30 to 90 mg·L^−1^, *C*_i_ was lower than that in the control group. When the concentration reached or exceeded 120 mg·L^−1^, *C*_i_ showed no significant difference compared to the control. Both *T*_r_ and *G*_s_ in sunflower leaves exhibited a decreasing trend with increasing m-cresol concentration and were significantly different from the control (*p* < 0.05). *P*_n_ decreased gradually with higher m-cresol levels, declining significantly to 55% of the control at 30 mg·L^−1^ and to 33.5% at 60 mg·L^−1^. Under m-cresol stress, water use efficiency (WUE) initially increased and then decreased; light energy utilization efficiency (LUE) continued to decrease. The results indicate that under low-concentration m-cresol stress, the reduction in sunflower photosynthesis is primarily regulated by stomatal factors; whereas under high-concentration stress, photosynthetic inhibition is driven by stomatal limitation.

### 3.2. Effects of M-Cresol Stress on Chlorophyll Fluorescence Parameters of Sunflower

**Figure 2:**
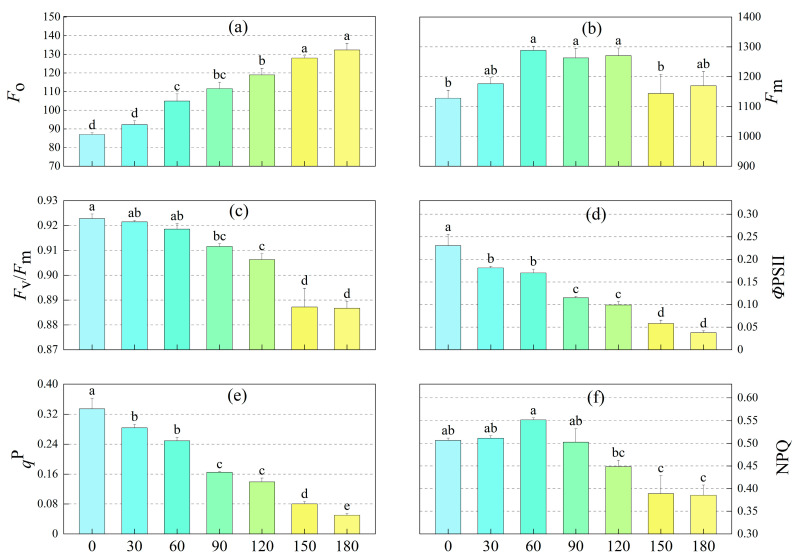
Chlorophyll fluorescence parameters of leaves of sunflower with m-cresol concentration. (**a**) Minimal fluorescence yield of the dark-adapted state (*F*_o_); (**b**) Maximum fluorescence yield (*F*_m_); (**c**) Maximal quantum yield of PSII photochemistry (*F*_v_/*F*_m_); (**d**) Effective quantum yield of PSII photochemistry (*Φ*PSII); (**e**) Photochemical quenching coefficient (*q*^P^); (**f**) Non-photochemical quenching (NPQ). Note: Different colors represent different concentrations of m-cresol; Different lowercase letters mean a significant difference among treatments at *p* < 0.05.

According to Figure 2, the chlorophyll fluorescence parameters of sunflower exhibited distinct response patterns under m-cresol stress. Minimal fluorescence yield of the dark-adapted state (*F*_o_) increased with rising m-cresol concentrations. At concentrations of 60, 90, 120, 150, and 180 mg·L^−1^, *F*_o_ values were 1.21, 1.28, 1.37, 1.47, and 1.52 times higher, respectively, than those in the control group (0 mg·L^−1^) (*p* < 0.05). This indicates that m-cresol interferes with the antenna pigment system or the original state of the PSII reaction center, with stronger disruption at higher concentrations. The maximum fluorescence (*F*_m_) was significantly higher in the m-cresol concentration range of 60–120 mg·L^−1^ compared to the control (*p* < 0.05), whereas no significant differences were observed between the other treatment groups and the control.

The maximum photochemical efficiency (*F*_v_/*F*_m_), the actual photochemical efficiency (*Φ*PSII), and the photochemical quenching coefficient (*q*^P^) all decreased with increasing m-cresol concentration (Figure 2c–e), indicating that m-cresol reduces the ability of the PSII system to convert light energy into chemical energy by inhibiting photosynthetic electron transport in sunflower leaves.

When the m-cresol concentration ranged from 0 to 60 mg·L^−1^, the non-photochemical quenching (NPQ) increased with rising m-cresol levels (Figure 2f). However, when the m-cresol concentration exceeded 90 mg·L^−1^, NPQ gradually decreased with further increases in concentration. This suggests that under low-concentration m-cresol stress, sunflowers can protect the photosynthetic system through thermal dissipation of excess light energy as a stress response.

Overall, the effect of m-cresol on the photosynthetic system of sunflowers was concentration-dependent. At m-cresol concentrations of 0–60 mg·L^−1^, sunflowers could mitigate damage by enhancing thermal dissipation. However, when the m-cresol concentration exceeded 90 mg·L^−1^, the photosynthetic system became impaired, leading to disruptions in electron transport. At this stage, the sunflowers could no longer counteract the stress through self-regulation (photoprotection), indicating severe damage to their physiological functions.

As shown in Table 1, with increasing m-cresol concentration, the PSII excitation energy partition coefficient (β), the fraction of absorbed light energy allocated to non-photochemical dissipation (Ex), and the quantum yield of non-regulated energy dissipation [Y(NO)] all increased significantly. In contrast, the quantum yield of regulated energy dissipation [Y(NPQ)] and the fraction of energy dissipated as heat through the antenna (D) first increased and then decreased. Conversely, the PSI excitation energy partition coefficient (α) and the fraction of absorbed light energy used for photochemistry (P) both decreased significantly.

**Figure 3:**
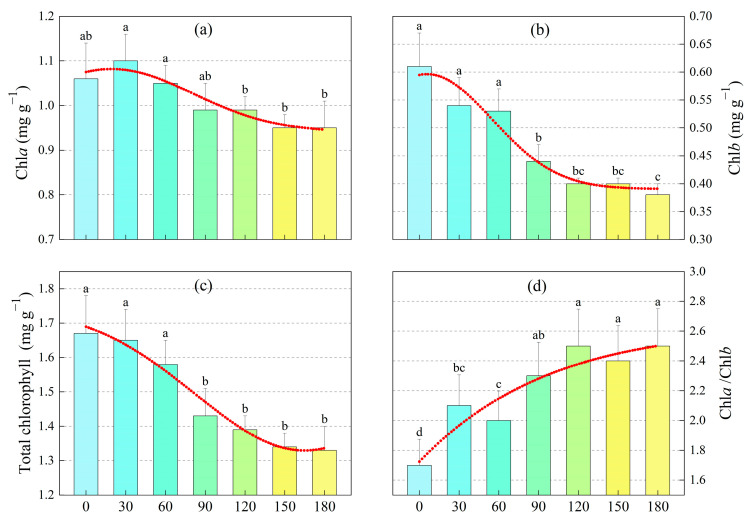
Changes in chlorophyll content in sunflowers under m-cresol trees. (**a**) chlorophyll a (Chl*a*); (**b**) chlorophyll b (Chl*b*); (**c**) total chlorophyll concentration; (**d**) chlorophyll a/Chlorophyll b (Chl*a*/Chl*b*). Note: The red line indicates the trend lines of Chl*a*, Chl*b*, total chlorophyll, and Chl*a*/Chl*b* with increasing m-cresol concentration; Different colors represent different concentrations of m-cresol; Different lowercase letters mean a significant difference among treatments at *p* < 0.05.

The chlorophyll a (Chl*a*) content in sunflower leaves ranged from 0.9 to 1.2 mg·g^−1^ and showed little variation with increasing m-cresol concentration (Figure 3a), with no significant differences observed between treatments (*p* > 0.05). In contrast, chlorophyll b (Chl*b*) and total chlorophyll concentrations showed an overall decreasing trend as the m-cresol concentration increased (Figure 3b,c). However, at higher m-cresol concentrations (120–180 mg·L^−1^), the Chl*b* and total chlorophyll content were significantly higher than those at lower concentrations (0–60 mg·L^−1^). The most substantial decrease in Chl*b* and total chlorophyll content was observed at 90 mg·L^−1^. The chlorophyll a/Chlorophyll b (Chl*a*/Chl*b*) exhibited an upward trend with increasing m-cresol concentration. The respective ratios at m-cresol concentrations of 0, 30, 60, 90, 120, 150, and 180 mg·L^−1^ were 1.7, 2.1, 2.0, 2.3, 2.5, 2.4, and 2.5.

### 3.3. Effects of M-Cresol Stress on Relative Growth Rate and M-Cresol Removal of Sunflower.

Overall, under m-cresol stress, chlorophyll a exhibited strong stability with minimal degradation. Chlorophyll *b* and total chlorophyll content remained relatively stable at low m-cresol concentrations (0–60 mg·L^−1^) but declined significantly at high concentrations (90–180 mg·L^−1^).

The relative growth rate (RGR) is a common metric in plant physiology and ecology for measuring the increase in plant biomass per unit time per unit of existing biomass. As shown in Table 2, the RGR of sunflowers decreased with increasing m-cresol concentration. Compared to the control group, the RGR values at m-cresol concentrations of 30, 60, 90, 120, 150, and 180 mg·L^−1^ were reduced by 28.0%, 64.0%, 96.0%, 192.0%, 216.0%, and 216.0%, respectively. At a concentration of 90 mg·L^−1^, the RGR of sunflowers was 0.001 (approaching zero), indicating nearly complete growth inhibition. When the m-cresol concentration reached 120–180 mg·L^−1^, the RGR values fell below zero, suggesting negative growth (i.e., biomass loss) in the plants. These results demonstrate that m-cresol inhibits sunflower growth, with the inhibitory effect intensifying at higher concentrations. Specifically, at concentrations ≥ 120 mg·L^−1^, sunflower growth not only ceased but also shifted toward progressive deterioration, eventually leading to plant mortality.

The removal rate of m-cresol decreased as its concentration increased. At an m-cresol concentration of 30 mg·L^−1^, the removal rate by sunflowers was the highest, reaching 84.91%. In contrast, at 180 mg·L^−1^, the removal rate was only 11.84%. This indicates that sunflowers are more effective at degrading m-cresol at low concentrations (0–60 mg·L^−1^).

## 4. Discussion

### 4.1. Photosynthetic Characteristics of Sunflower

Photosynthesis, the basis for normal plant growth and metabolic activity, is impeded by abiotic stress [36]. A decline in the net photosynthetic rate (*P*_n_) is usually attributed to both stomatal and non-stomatal limitations [37]. Stomatal limitation often results from reduced stomatal conductance (*G*_s_) caused by water stress, which restricts CO_2_ entry into leaves. Non-stomatal limitation typically arises from damage to the chloroplast structure, impairing photosynthetic activity [38]. In this study, increasing m-cresol concentration led to a decrease in *G*_s_ of sunflower leaves, inducing gradual stomatal closure and elevated CO_2_ diffusion resistance [39]. Consequently, the intercellular CO_2_ concentration (*C*_i_) could not be effectively replenished; it initially decreased and then increased. This weakening of photosynthetic capacity resulted in a reduced *P*_n_, indicating that photosynthesis in sunflower leaves was inhibited by m-cresol stress. According to Farquhar and Sharkey [40], the primary reason for the decrease in *P*_n_ at m-cresol concentrations ≤ 90 mg·L^−1^ is stomatal limitation. In contrast, at concentrations ≥ 120 mg·L^−1^, non-stomatal factors become the main cause of the *P*_n_ decline. A critical shift from stomatal to non-stomatal limitations appears to occur in sunflowers between 90 and 120 mg·L^−1^ of m-cresol. At these higher concentrations, m-cresol likely disrupts chlorophyll synthesis in sunflower seedling leaves, leading to a significant reduction in *P*_n_ [41]. This contrasts with findings for aniline stress, where the net photosynthetic rate of sunflower at 100 mg·L^−1^ aniline was reported to be 1.4 times higher than that of the control [23].

In the present experiment, the *P*_n_ of sunflower was significantly reduced at m-cresol concentrations of 90–120 mg·L^−1^, reaching only 21–31% of the control value. Some studies have proposed a 50% decrease in *P*_n_ as a tolerance threshold for adversity stress in plants [23,42]. Using *P*_n_ as an indicator, the tolerance index (TI) and toxicity factor were calculated for different m-cresol concentrations (X). The m-cresol concentration corresponding to a 50% reduction in *P*_n_ was calculated to be 51.9 mg·L^−1^ based on the regression equation (*P*_n_ = 0.0005X^2^ − 0.1659X + 14.532R^2^ = 0.9501). Thus, 51.9 mg·L^−1^ is recommended as the upper limit for using sunflowers in phytoremediation of m-cresol-contaminated wastewater.

Under normal conditions, light use efficiency (LUE) and water use efficiency (WUE) are in dynamic equilibrium. Under stress conditions, however, internal water and light energy utilization are altered. In sunflowers under m-cresol stress, WUE initially increased and then decreased. We speculate that the plant first allocates energy to maintain water use processes, then actively reduces the transpiration rate (*T*_r_) to prevent excessive water loss as m-cresol concentration increases. Simultaneously, water uptake and transport are inhibited, leading to an imbalance in water balance and a subsequent decrease in WUE, which disrupts normal water metabolism [43]. Conversely, LUE decreased significantly with increasing m-cresol concentration, likely because sunflowers under m-cresol stress adopt a trade-off strategy of prioritizing WUE at the expense of LUE. 

### 4.2. Chlorophyll Fluorescence Parameters of Sunflower

Chlorophyll fluorescence measurements are widely used to study photosynthetic function and serve as a valuable tool for investigating plant responses to environmental stress [44,45,46]. A well-documented and important relationship exists between photosynthesis and chlorophyll (Chl*a*) fluorescence [47]. Therefore, chlorophyll fluorescence is widely employed as a reliable method for assessing photosynthetic efficiency, particularly that of PSII [48,49].

*F*_o_ represents the fluorescence level when the PSII reaction center is fully open. Its magnitude reflects the condition of the PSII reaction center, and an increase in *F*_o_ may indicate damage or reversible inactivation of this center [50]. A high value of Y(NO) reflects the plant’s inability to protect itself from damage caused by excessive excitation energy [51]. In this study, as the m-cresol concentration increased, *F*_o_ increased, NPQ initially increased and then decreased, while *F*_o_ and Y(NO) continued to rise as NPQ declined. This suggests that the PSII super complex was protected by regulated energy dissipation during the initial gradual increase in m-cresol concentration. Low concentrations of m-cresol significantly reduced *q*^P^ while increasing NPQ and Y(NPQ) in sunflower leaves, indicating that m-cresol stress inhibited the photosynthetic mechanism and reduced the photochemical efficiency of PSII. This triggered the dissipation of excess excitation energy to sustain photosynthetic function. However, NPQ and Y(NPQ) tended to decrease when m-cresol concentrations exceeded 90 mg·L^−1^. The stress also led to an imbalance in the distribution of excitation energy between the two photosystems (α decreased while β increased). The significant increase in excitation energy allocated to PSII (β) can cause reversible deactivation of reaction centers and even damage to PSII and thylakoid membrane structures, thereby impeding photosynthetic electron transfer and limiting photosynthetic efficiency. Specifically, a decrease in the value of P (the fraction of energy used for photochemistry) increases the accumulation of excess light energy in PSII. This elevates the value of D (the fraction of energy dissipated by antenna pigments), which depends on NPQ dissipation [52]. When the PSII reaction center closes or becomes inactivated, and the excitation energy delivered to it cannot be used for photochemistry, the increase in the Ex fraction induces the multiplication of reactive oxygen species. This directly damages chlorophyll structural proteins in the reaction center, leading to a decrease in *Φ*PSII. In brief, low levels of m-cresol stress activate the photodamage defense mechanism in sunflower leaves. The light energy absorbed by PSII is increasingly diverted to thermal dissipation via antenna pigments to alleviate damage to the PSII reaction center machinery. In contrast, high levels of m-cresol stress disrupt this photodamage defense system, leading to the destruction or reversible inactivation of the PSII reaction center. *F*ᵥ/*F*ₘ reflects the primary light energy capture efficiency of the open PSII reaction center [21]. At m-cresol concentrations up to 90 mg·L^−1^, the *F*ᵥ/*F*ₘ of sunflower leaves was significantly different from that of the control [53]. *q*^P^ reflects the proportion of open PSII reaction centers; a higher value indicates greater electron transfer activity in PSII [54]. In the present study, the decreasing trend in *q*^P^ indicated that a reduction in the number of open PSII centers partly led to the decrease in *P*_n_ under m-cresol stress.

### 4.3. Effect of M-Cresol on Chlorophyll Content of Sunflower

Chloroplast pigments can transfer or store the light energy absorbed from sunlight into chemical energy. Their content indicates the photosynthetic intensity in plant leaves and effectively reflects the photosynthetic capacity [55]. In sunflower seedlings under m-cresol stress, the contents of Chl*a*, Chl*b*, and total chlorophyll (Chl*a* + Chl*b*) decreased significantly and gradually at concentrations of 60–90 mg·L^−1^ (*p* < 0.05). Concurrently, the limitation on photosynthesis in sunflower leaves shifted from stomatal to non-stomatal factors. It can be speculated that this shift may be directly related to the significant decrease in the accumulation of all chlorophyll types. Organic pollutants, such as oil and its derivatives, can form an impermeable layer on the root surface, impeding the uptake of water and mineral salts from the soil [56]. These pollutants significantly inhibit photosynthetic efficiency by disrupting the chloroplast ultrastructure and inhibiting chlorophyll biosynthesis. Therefore, we hypothesize that under m-cresol stress, chlorophyll synthesis was inhibited and chlorophyll degradation was accelerated in sunflowers.

### 4.4. Decontamination Effect of Sunflower on M-Cresol

Changes in plant growth morphology are the most visible indicators of stress response [57]. In the study, the relative growth rate (RGR) of sunflowers decreased significantly with increasing m-cresol concentration. The removal efficiency of plants for organic pollutants can be determined by measuring the residual pollutant concentration in contaminated water. Jing Tao found that sunflowers effectively remediate aniline-contaminated water, achieving a removal rate of 80.97% for wastewater containing 100 mg·L^−1^ aniline [23]. Similarly, Fu reported that *Acorus calamus* removed 83.59% of phenol from a 100 mg·L^−1^ solution within 21 days of treatment [14]. In this study, under low-concentration m-cresol stress, although sunflower growth was affected to some extent, the plants maintained a relatively high m-cresol removal capacity. However, when the m-cresol concentration exceeded 90 mg·L^−1^, sunflower growth was severely inhibited, and their m-cresol removal capacity also decreased significantly.

The plant antioxidant enzyme system is a crucial defense mechanism against oxidative stress. Superoxide dismutase, peroxidase, and catalase constitute the core components of this system, playing vital roles in maintaining cellular redox balance [58]. However, their specific roles in the sunflower’s phytoremediation of m-cresol warrant further investigation.

## 5. Conclusions

Low-concentration m-cresol restricts the entry of CO_2_ into leaves by reducing stomatal conductance, leading to a decrease in intercellular CO_2_ concentration and a subsequent decline in the net photosynthetic rate. At this stage, photosynthetic inhibition is primarily governed by stomatal limitation. In contrast, high-concentration m-cresol damages chloroplast structure and function, causing degradation of Chl*b* and irreversible harm to the reaction center of photosystem II. Under such conditions, photosynthetic inhibition is mainly due to non-stomatal limitation. Regression analysis determined that a m-cresol concentration of 51.9 mg·L^−1^ results in a 50% reduction in the net photosynthetic rate of sunflowers. This concentration can thus be regarded as the safe upper limit for sunflower-mediated remediation of m-cresol wastewater.

Under low-concentration m-cresol stress (0–60 mg·L^−1^), sunflowers dissipate excess light energy absorbed by PSII as heat by enhancing non-photochemical quenching (NPQ) and regulating the quantum yield of regulated energy dissipation [Y(NPQ)], thereby protecting the PSII reaction center from damage. However, when the concentration exceeds 90 mg·L^−1^, this photoprotection mechanism fails. NPQ and Y(NPQ) decrease, while [Y(NO)] increases, leading to damage in the PSII reaction center and obstruction of electron transfer.

Low-concentration m-cresol (0–60 mg·L^−1^) only slightly inhibits sunflower growth, allowing the plants to maintain a certain level of biomass accumulation. When the concentration reaches or exceeds 120 mg·L^−1^, the RGR becomes negative, resulting in biomass loss and ultimately posing a risk of plant death.

In conclusion, as a plant with both economic value and environmental adaptability, sunflowers demonstrate strong remediation potential for wastewater contaminated with low-concentration m-cresol and can be effectively applied in the treatment of m-cresol wastewater with concentrations ≤ 51.9 mg·L^−1^.

## Figures and Tables

**Table 1:** Distribution of excitation energy between two photosystems and PSII luminous energy.

Treatment	Y (NPQ)	Y (NO)	f	α	β	P	D	Ex
0	0.45 ± 0.021 b	0.32 ± 0.021 e	0.33 ± 0.031 a	0.25 ± 0.024 a	0.76 ± 0.015 e	0.23 ± 0.015 a	0.31 ± 0.027 b	0.46 ± 0.016 e
30	0.48 ± 0.012 a	0.34 ± 0.012 e	0.28 ± 0.013 b	0.22 ± 0.012 b	0.78 ± 0.006 d	0.18 ± 0.004 b	0.36 ± 0.008 a	0.46 ± 0.008 e
60	0.45 ± 0.005 b	0.38 ± 0.013 d	0.25 ± 0.014 b	0.20 ± 0.011 b	0.80 ± 0.007 d	0.17 ± 0.007 b	0.31 ± 0.007 b	0.51 ± 0.004 d
90	0.41 ± 0.012 c	0.48 ± 0.013 c	0.17 ± 0.004 c	0.14 ± 0.003 c	0.86 ± 0.004 c	0.12 ± 0.004 c	0.30 ± 0.008 b	0.58 ± 0.005 c
120	0.37 ± 0.011 d	0.53 ± 0.021 b	0.14 ± 0.009 c	0.12 ± 0.010 c	0.88 ± 0.010 c	0.10 ± 0.006 c	0.29 ± 0.006 b	0.61 ± 0.008 c
150	0.31 ± 0.022 e	0.64 ± 0.024 a	0.08 ± 0.007 d	0.07 ± 0.008 d	0.93 ± 0.009 b	0.06 ± 0.007 d	0.28 ± 0.025 b	0.66 ± 0.030 b
180	0.29 ± 0.009 e	0.67 ± 0.006 a	0.05 ± 0.004 d	0.04 ± 0.003 e	0.95 ± 0.004 a	0.04 ± 0.002 d	0.24 ± 0.008 c	0.72 ± 0.007 a

Note: Different lowercase letters in the same column mean a significant difference among treatments at *p* < 0.05

**Table 2:** Changes in sunflower Relative growth rate and removal percentage of m-cresol.

Concentration of M-Cresol (mg L^−1^)	Relative Growth Rate (RGR)	Percent Removal (%)
0	0.025 ± 0.0024	-
30	0.018 ± 0.0012	84.91
60	0.009 ± 0.0011	57.28
90	0.001 ± 0.0009	40.11
120	−0.023 ± 0.0021	21.45
150	−0.029 ± 0.0029	15.59
180	−0.029 ± 0.0034	11.84

## Data Availability

Data are contained within the article.
